# Microbial Community Redundancy and Resilience Underpins High-Rate Anaerobic Treatment of Dairy-Processing Wastewater at Ambient Temperatures

**DOI:** 10.3389/fbioe.2020.00192

**Published:** 2020-03-13

**Authors:** Lara M. Paulo, Juan Castilla-Archilla, Javier Ramiro-Garcia, José Antonio Escamez-Picón, Dermot Hughes, Thérèse Mahony, Michael Murray, Paul Wilmes, Vincent O'Flaherty

**Affiliations:** ^1^Microbiology, School of Natural Sciences and Ryan Institute, NUI Galway, Galway, Ireland; ^2^Dairy Processing Technology Centre (DPTC), Limerick, Ireland; ^3^Luxembourg Centre for Systems Biomedicine, University of Luxembourg, Esch-sur-Alzette, Luxembourg; ^4^NVP Energy Ltd., Galway Technology & Business Centre, Galway, Ireland

**Keywords:** ambient temperature anaerobic digestion, biogas, dairy wastewater, microbial community, perturbations

## Abstract

High-rate anaerobic digestion (AD) is a reliable, efficient process to treat wastewaters and is often operated at temperatures exceeding 30°C, involving energy consumption of biogas in temperate regions, where wastewaters are often discharged at variable temperatures generally below 20°C. High-rate ambient temperature AD, without temperature control, is an economically attractive alternative that has been proven to be feasible at laboratory-scale. In this study, an ambient temperature pilot scale anaerobic reactor (2 m^3^) was employed to treat real dairy wastewater *in situ* at a milk processing plant, at organic loading rates of 1.3 ± 0.6 to 10.6 ± 3.7 kg COD/m^3^/day and hydraulic retention times (HRT) ranging from 36 to 6 h. Consistent high levels of COD removal efficiencies, ranging from 50 to 70% for total COD removal and 70 to 84% for soluble COD removal, were achieved during the trial. Within the reactor biomass, stable active archaeal populations were observed, consisting mainly of *Methanothrix* (previously *Methanosaeta*) species, which represented up to 47% of the relative abundant active species in the reactor. The decrease in HRT, combined with increases in the loading rate had a clear effect on shaping the structure and composition of the bacterial fraction of the microbial community, however, without affecting reactor performance. On the other hand, perturbances in influent pH had a strong impact, especially when pH went higher than 8.5, inducing shifts in the microbial community composition and, in some cases, affecting negatively the performance of the reactor in terms of COD removal and biogas methane content. For example, the main pH shock led to a drop in the methane content to 15%, COD removals decreased to 0%, while the archaeal population decreased to ~11% both at DNA and cDNA levels. Functional redundancy in the microbial community underpinned stable reactor performance and rapid reactor recovery after perturbations.

## Introduction

The high demand milk and milk products has led to an increase in dairy production globally. In the EU, since the removal of milk production quotas in 2015, the dairy industry has undergone rapid growth (Gil-Pulido et al., 2018). Dairy plants produce large volumes of wastewater; it is estimated that 1–2 m^3^ of wastewater is produced per m^3^ of manufactured milk (Quaiser and Bitter, 2016; Slavov, 2017). These wastewaters are characterized by high organic load and nutrient composition (Demirel et al., 2005; Lateef et al., 2013; Gil-Pulido et al., 2018). Several approaches, including physical-chemical and biological processes, are applied to treat dairy wastewaters. However, physico-chemical processes present high reagent costs and low chemical oxygen demand (COD) removals, leading to the favoring of biological processes (Demirel et al., 2005; Gil-Pulido et al., 2018). High-rate anaerobic digestion (AD) is an efficient and well-established biological process to treat wastes and wastewaters. By comparison with aerobic processes, AD presents several advantages, including lower quantities of generated waste-sludge, smaller reactor volumes and the production of a renewable fuel—biogas methane, that can displace fossil natural gas to produce heat and energy (McKeown et al., 2012). High-rate AD technology relies on the retention of high levels of active microorganisms within the system. This is achieved by the immobilization of the microbes on a support material or by the formation of granules (McKeown et al., 2012). These reactors tolerate short HRT (1–24 h) and high organic loading rates (up to 100 kg COD/m^3^/day; McKeown et al., 2012).

In general, AD systems are operated under mesophilic (30–37°C) or thermophilic conditions (45–55°C) ensure maximum microbial growth and reaction rates. However, dairy wastewaters are often discharged at lower temperatures (~17–18°C in winter and 22–25°C in summer (Slavov, 2017). If AD is to be used to treat this wastewater at high-rates, heating such large volumes of wastewater for this purpose is economically and environmentally unfavorable.

AD at ambient or low temperatures (<20°C) (Lt-AD) is an economically attractive alternative. Research on the treatment of domestic sewage at low temperature reported promising results with good COD removals being reported: up to 87% in two hybrid reactors with HRT of 8 h (Elmitwalli et al., 1999) and up to 81% in a two-step system consisting of an anaerobic filter and an anaerobic hybrid operated at HRT 4 h (Elmitwalli et al., 2002). Despite that, many limitations were associated with Lt-AD and thus it was initially considered unfeasible for many complex industrial streams including those produced by dairy-processing (McKeown et al., 2012). A better understanding of the nature and limitations of anaerobic microbial consortia and improvements in process configuration has suggested, however, that the process was feasible and suitable for scale-up trials (McHugh et al., 2006; Akila and Chandra, 2007; Enright et al., 2009; McKeown et al., 2009). To our knowledge, this is the first report of pilot-scale, high-rate, AD of dairy-processing wastewater.

The AD process relies on the degradation of organic matter by a network of microorganisms presenting diverse nutritional requirements and physiological characteristics (Shah, 2014). These microorganisms also present different responses to environmental stresses, such as temperature, pH variations, substrate composition/concentration or the presence of inhibitory or toxic compounds (Shah, 2014; Venkiteshwaran et al., 2015). Several studies have focused on the development of microbial communities in laboratory-scale AD bioreactors operated at >35°C to lower temperature conditions, with special focus on the methanogenic portion of communities (Enright et al., 2009; McKeown et al., 2009; O'Reilly et al., 2009; Abram et al., 2011; Bandara et al., 2012; Zhang et al., 2012; Gunnigle et al., 2015a,b; Keating et al., 2018). Nevertheless, very little is known about the potential development of such communities at pilot and full-scale, or how they respond under environmental stresses, such as variations in operational parameters.

The main goal of this study was thus to explore the relationships between microbial community structure and reactor performance in a pilot scale (2 m^3^) high-rate AD reactor, operated at ambient temperature, during treatment of industrial dairy processing wastewater.

## Materials and Methods

### Pilot-Scale Reactor Design and Operation

The reactor was a stainless-steel vessel mounted on a transportable steel frame, designed in the configuration described by Hughes et al. (2011) with a total volume of 2 m^3^ and an active volume of 1.8 m^3^ (Figures S1, S2). In summary, the reactor was a hybrid of a sludge blanket reactor divided in two different main parts, the first corresponding to a granular sludge system in the lower section of the reactor, and a second part corresponding to an anaerobic filter located on the top section. The reactor was seeded with anaerobic granular sludge from an industrial UASB for the treatment of wastewater in a slaughterhouse plant. The trial was performed at a wastewater dairy processing plant in the Republic of Ireland. The wastewater used in this trial was taken from the dairy processing plant effluent, after the bulk of the fats, oils and grease (FOG) were separated by dissolved air flotation. Prior to entering the reactor, the wastewater was first diverted into a homogenization tank of 1 m^3^, where the pH was maintained at 7.5 ± 0.2 for the inlet flow using a pH controller Alpha pH 200 (Thermo Scientific), connected to two 323S Watson-Marlow (UK) pumps for addition of NaOH or HCl as required. The influent was then pumped into the pilot reactor from the homogenization tank using a 620S Watson-Marlow (UK) pump. The reactor was operated with a constant liquid up-flow velocity of 1.8 m/h by recirculation of reactor effluent using a 620S Watson-Marlow (UK) pump. No temperature control was applied to the wastewater or to the reactor vessel. The in-reactor temperature fluctuated between 21.9 and 30.1°C during the trial (Figure S2). The trial was carried out over a period of 291 days, divided into 7 different phases (Table 1). During the course of the trial, the applied hydraulic retention time (HRT) was reduced from 36 h (Phase 1) to 6 h (Phase 7).

**Table 1:** Summary of the reactor trial.

**Phase**	**Phase 1**	**Phase 2**	**Phase 3**	**Phase 4**	**Phase 5**	**Phase 6**	**Phase 7**
HRT (h)	36	30	24	18	12	9	6
Influent total COD (kg/m^3^)	2.2 ± 0.6	1.7 ± 0.8	2.1 ± 0.8	2.1 ± 0.6	2.1 ± 1.0	2.5 ± 0.6	2.7 ± 0.9
Effluent total COD (kg/m^3^)	0.7 ± 0.2	0.7 ± 0.3	0.5 ± 0.2	0.6 ± 0.2	0.9 ± 0.5	1.3 ± 0.9	1.1 ± 0.2
Total COD Removal (%)	70.7 ± 14.2	52.9 ± 24.4	70 ± 14.2	67.8 ± 12.3	55.1 ± 14.4	49.3 ± 23.5	52.5 ± 14.3
Influent soluble COD (kg/m^3^)	519 ± 38	578 ± 305	1024 ± 560	936 ± 398	1117 ± 539	1400 ± 409	1725 ± 677
Effluent soluble COD (kg/m^3^)	0.07 ± 0.01	0.1 ± 0.08	0.1 ± 0.04	0.1 ± 0.10	0.2 ± 0.2	0.5 ± 0.7	0.5 ± 0.3
Soluble COD Removal (%)	82.2 ± 10.1	74.3 ± 23.1	83.6 ± 15.7	84.3 ± 7.4	82.4 ± 14.8	70.9 ± 29.6	83.2 ± 13.2
Loading rate (kg/m^3^.day)	1.5 ± 0.4	1.3 ± 0.6	2.1 ± 0.8	2.7 ± 0.8	3.9 ± 0.2	6.7 ± 1.5	10.6 ± 3.7
pH	7.5 ± 0.3	8.0 ± 0.7	7.6 ± 0.3	7.6 ± 0.2	7.8 ± 0.6	8.2 ± 0.7	8.1 ±.5
Average methane content (%)	83.2 ± 3.3	85.5 ± 6.1	85.4 ± 8.9	83.1 ± 5.6	80.1 ± 5.8	73.3 ± 29.5	89.6 ± 3.2

### Analytical Methods

Total and Soluble COD were measured using a kit of Reagecon (Shannon, Ireland) test medium range COD vials (0–1,500 mg/L), according to the manufacturer's instructions. The methane content in the biogas was determined by gas chromatography (CP-3800 Varian Inc., Walnut Creek, CA) according to standard methods (APHA, 2005). The FOG were measured a Wilks Infracal 2 HATR/ATR-SP (USA), according to the standard method in the EPA 1664 (USEPA, 2010).

### Microbial Community Analysis

#### Sample Collection and DNA/RNA Extraction

Granular sludge samples were periodically withdrawn from the reactor via a sampling port located close to the base of the unit. The samples were instantly frozen in liquid nitrogen and stored at −80°C until processing for DNA/RNA extraction. Granules were crushed in liquid nitrogen using a pestle and mortar until is a fine powder. Approximately 0.1 g of granule's powder was weighted in sterile 2 mL vials containing zirconia beads, 500 μL of 1% CTAB buffer and 1 ml of Phenol:Chloroform:Isoamyl alcohol (25:24:1). Cells were disrupted using a VelociRuptor Microtube Homogenizer for two cycles of 60 s each. For each time point, DNA/RNA were extracted in triplicate according to the protocol described by Griffiths et al. (2000) with the modification of Thorn et al. (2018). DNA/RNA quality was assessed using 1% (w/v) agarose gel containing 1× SYBR® Safe (Invitrogen, Carlsbad, CA). RNA was treated with Turbo-DNA *free*^TM^ Kit (Thermo Fisher Scientific, Whaltam, MA) to remove contaminating DNA. RNA and DNA concentrations were determined using a Qubit Fluorometer (Thermo Fisher Scientific).

#### Library Preparation

Reverse transcription was performed using Primers for cDNA Synthesis (Thermo Fisher Scientific) and SuperScript™ III Reverse Transcriptase (Thermo Fisher Scientific).

DNA and cDNA were amplified by targeting the V4 region of the 16S rRNA using the primers 515f (5′-GTGCCAGCMGCCGCGGTAA) and 806r (5′-GGACTACHVGGGTWTCTAAT). Analysis of the primer coverage can be found in the Supplementary Material. The amplicons were generated using one-step PCR. For this, 70-barcoded primers were used as described by Ramiro-Garcia et al. (2018). The 10–20 ng of DNA was used as template in the PCR reaction (50 μL), which contained 10 μL HF buffer (Thermo Fisher Scientific), 1 μL dNTP Mix (10 mM; Bioline, London, UK), 1 U of Phusion Hot Start II DNA Polymerase (Thermo Fisher Scientific), 500 nM of each barcoded primer. PCRs were performed with an Alpha cycler 1 (PCRmax, Staffordshire, UK) using an adaptation of the cycling conditions of Caporaso et al. (2012). The cycling conditions consisted of an initial denaturation at 98°C for 3 min, 25 cycles of: 98°C for 10 s, 50°C for 20 s, and 72°C for 20 s, and a final extension at 72 °C for 10 min. The size of the PCR products (~330 bp) was confirmed by agarose gel electrophoresis using 5 μL of the amplification-reaction mixture on a 1% (w/v) agarose gel. For each sample, the PCRs were done in duplicate and pooled together before purification. The pooled PCR products were purified with HighPrep^TM^ (Magbio Genomics, Gaithersburg, MD, United States) using 20 μL of Nuclease Free Water (Bioline) for elution and then quantified using a Qubit (Thermo Fisher Scientific) in combination with the dsDNA HS Assay Kit (Thermo Fisher Scientific). The purified products were mixed together in equimolar amounts to create two library pools, one for DNA and one for cDNA, and sent for sequencing on the Illumina Hiseq 2000 platform (GATC Biotech AG, Konstanz, Germany). Sequence data have been deposited in European Nucleotide Archive, accession number [PRJEB29981].

#### Bioinformatics and Statistical Analysis

Data was analyzed using NG-Tax (Ramiro-Garcia et al., 2018), a validated pipeline for 16S rRNA analysis, under default parameters. Independently for each sample, most abundant sequences (>0.1%) were selected as ASV collecting 9.485.867 reads for all samples. To correct for sequencing errors, the remaining reads were clustered against those ASVs allowing one mismatch, reaching a total of 12.862.549 reads. The database used for the analysis was Silva 128 and the primers covered 98.4% of the 1.783.650 Bacteria and Archaea phylotypes included. AD specific databases like MiDAS (McIlroy et al., 2017) may improve the accuracy of the taxonomical assignments by reducing the number of possible candidates at the expense of generating misannotations due to its lack of completeness. Since the average accuracy for the ASVs in this study was very high (97.3%), with 76.7% of the ASVs having an accuracy of 100% (meaning all hits belong to the same genera) specific databases were not used. Alpha diversity was calculated and plotted using the R packages picante (Kembel et al., 2010) and ggplot2 (Wickham, 2016). Beta diversity and Constrained Analysis of Principal Coordinates (CAP) under the model ~ HRT + pH were performed using phyloseq (McMurdie and Holmes, 2013) via the capscale (Oksanen, 2012) package.

## Results

### Reactor Performance

**Figure 1:**
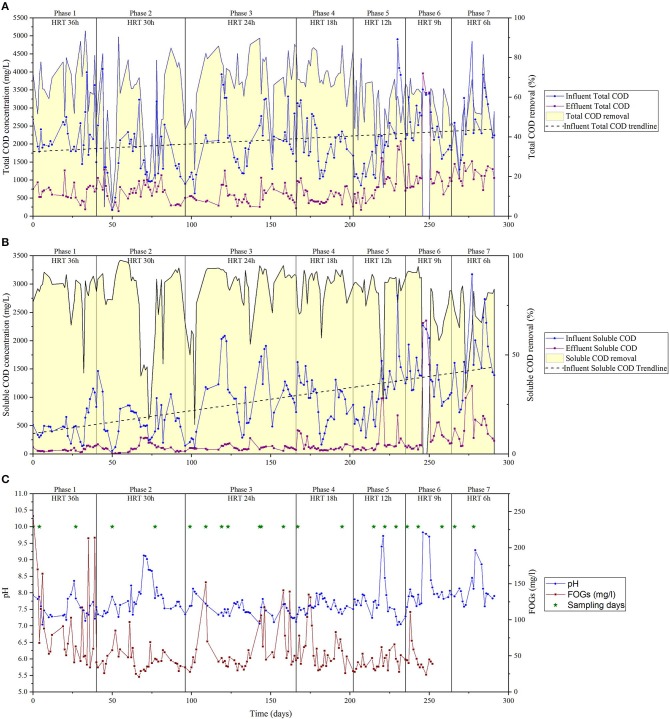
Total and Soluble COD in the influent and effluent and COD removal **(A,B)** and FOGs, pH **(C)** over time. FOGs and pH data were collected for the inlet of the reactor. Biomass sampling dates are also indicated **(C)**.

**Figure 2:**
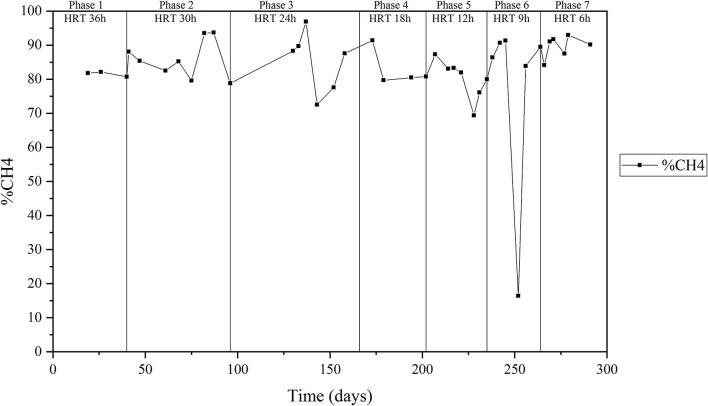
Average methane content in the biogas over the trial.

The HRT applied to the reactor was decreased stepwise from 36 to 6 h in seven phases. The average total and soluble influent COD during the trial fluctuated greatly (0.20 and 4.9 kg/m^3^ of total COD and between 0.05 and 3.1 kg/m^3^ for soluble COD), mainly due to changes in production processes of the factory (Table 1, Figures 1A,B). This corresponded to an organic loading rate of 1.3 ± 0.6 to 10.6 ± 3.7 kg COD/m^3^/day (Table 1). Total COD removal was between 49 and 71%, while the average soluble COD removal was more stable over the trial, fluctuating between 71 and 84.3%. A technical failure of the acid-addition pump resulted in a significant pH perturbation on day 246 that lasted until the pump was repaired on day 250, resulting in the pH of the reactor liquor increasing to >8.5. A number of less significant pH perturbations occurred on days 26, 220, 279 etc., arising from power supply interruptions, resulting in transient increase in pH to >8.5 for 1–2 days (Figure 1C). Low COD removal and low methane content was observed when the pH was above 8.5 (Figure 1C). This was especially so during days 246–251, when the reactor pH was 9.7–9.8 for four days, no COD removal was observed, and the methane content dropped to ~15%. Once the pump was operating again, COD removal rates recovered to values in the same magnitude as seen prior to the incident within 5 days (Figure 1B). However, the methane content required almost 15 days to reach the previous values. The methane content in the biogas during the whole trial averaged between 73.4 ± 29.5%, if excluding the values obtained during the pH shock (days 246 to 251) the overall methane content was 89.6 ± 3.2% (Figure 2). The FOG content in the inlet of the reactor along the trial was 60.5 ± 39.7 mg/L during the trial, with the lowest value of FOG corresponded to 21 mg/L and the highest value to 244 mg/L. No significant effect was observed in the reactor's performance due to increases in influent FOG concentrations.

### Microbial Community Analysis

**Figure 3:**
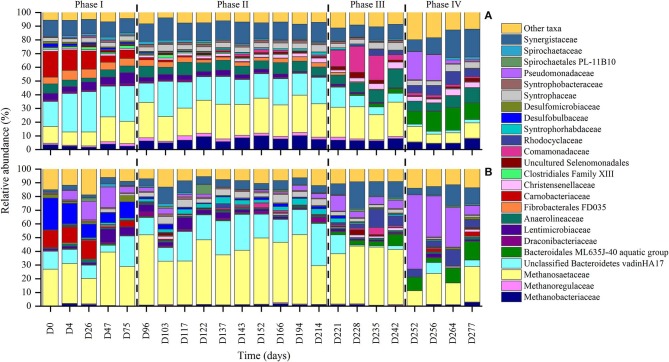
Relative abundances for the relevant taxonomic groups of the microbial communities for DNA **(A)** and cDNA **(B)**. The data represents, for each day, the average off the triplicates and a cut-off of 1% was applied.

The composition of the microbial community was analyzed at several time points over the trial (Figure 1C). The results showed a stable core of archaeal populations, both at DNA and cDNA level (Figure 3). Furthermore, a heat-map was constructed for the relevant taxonimcal groups and is provided in Figure S4. DNA-based data indicated that *Methanobacteriaceae* species (up to 10%), *Methanosaetaceae* species (up to 23%). Furthermore, cDNA results indicated that members of *Methanosaetaceae* species, with relative abundances up to 47%, were the active core of microbial community. Unclassified members of VadinHA17 (up to 29%), and unclassified members of *Synergistaceae* (up to 21%), were the most relatively abundant bacterial groups present.

Although there was a homogenization tank system in place prior to the AD system where the pH was controlled, the pH inside the AD system suffered periodic oscillations. Effects at microbial community level associated with pH perturbations were, however, only observed when pH values where higher than 8.5 (at days 70–75, 221, and 246–250). These pH shocks induced changes in the community, which can be divided into four phases (Figure 3).

In phase I (days 0–75), in addition to the core members, a high relative abundance of *Carnobacteriaceae* species where present both in DNA-based (up to 18%) and cDNA-based (up to 13%) datasets. Furthermore, cDNA-based analysis also revealed the high relative abundance of *Desulfobulbaceae* species (up to 22%). After the pH shock on days 70–75, both taxa were present as <1% of the community in relative abundance terms.

In phase II (days 96–214), the DNA-based data indicated an increase in *Methanosaetaceae* species from 8 to 13% (phase I) to 18–23%. At the cDNA level, there was an increase of unclassified members of Bacteroidetes' class vadinHA17, from 9–13% to 17–31%. This taxon had decreased in relative abundance to <2% by phase III.

In the third phase (days 221–242), an increase in unclassified members of the family *Comamonadaceae* was observed, from 0–2.5% to 12–20%, as well as an increase in the relative abundance of *Bacteroidales* ML635J-40, from <1% to 2–3.2%, at the DNA level. At the same time, a decrease in the relative abundance of unclassified Bacteroidetes vadinHA17 for <2% at cDNA level and ~6% at DNA level. During this phase, an increase in members from the *Rhodocyclaceae* family was observed, up to 4 and 13% in DNA-based and cDNA-based datasets, respectively.

**Figure 4:**
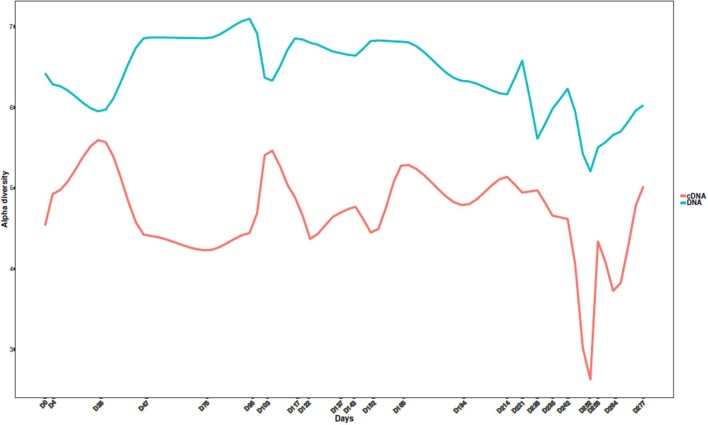
Alpha diversity variation over time.

Finally, in phase IV (days 252–277), a considerable reduction in the relative abundance of the Archaea, both at a DNA (from 25–35% to 10–11%) and a cDNA (from 35–50 to 11%) level could be observed due to the exposure to high pH levels for several days. However, the system was able to recover and 20 days after the pH shock, the relative abundance of active Archaea was ~30%. It was also observed that the relative abundance of *Bacteroidales* ML635J-40 increased up to 13.5 and 16% at the cDNA and DNA levels, respectively. Moreover, after the prolonged pH shock, an increase in members of *Pseudomonadaceae* family was observed, both in DNA-based (from <1% to ~20%) and in cDNA-based (from 5 to 50%) datasets. Following the return of pH values to ~7.5, the relative abundances of *Pseudomonadaceae* family decreased over time and had reverted to the same values as before the shock by day 277 (~1% for DNA and ~6% for cDNA; Figure 3). At the DNA level, a doubling of the relative abundances of unclassified members of the family *Synergistaceae* was observed. The prolonged pH shock (days 246–250) also affected the alpha diversity of the microbial community (Figure 4), which significantly decreased both at the DNA and cDNA level, while the shorter pH shocks showed no visible effect on diversity.

**Figure 5:**
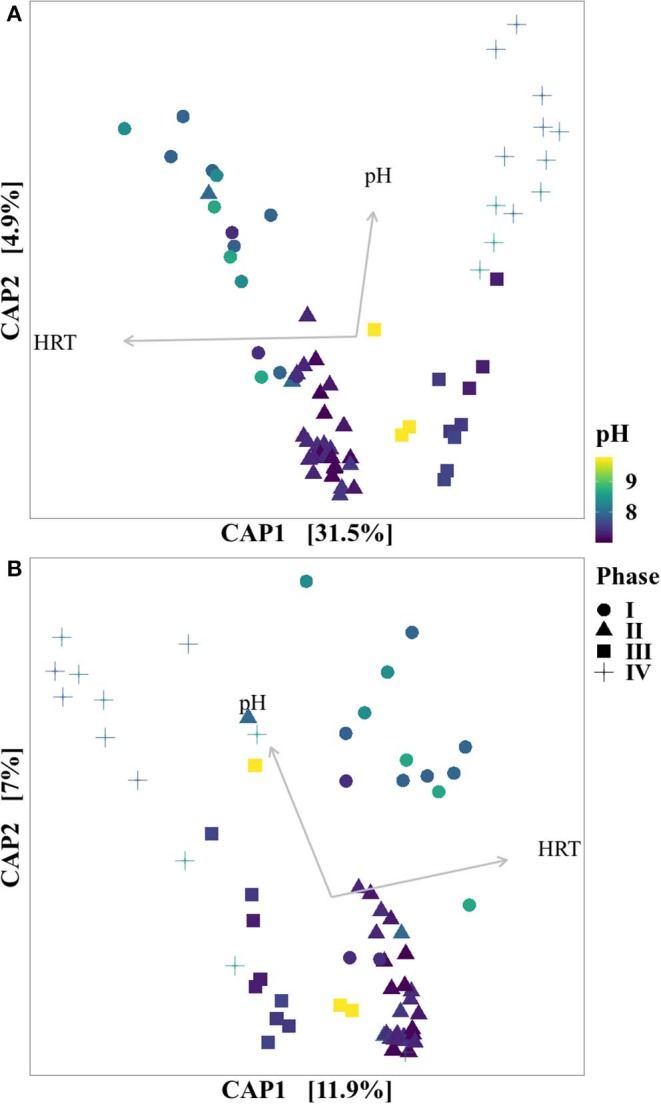
CAP plots for DNA **(A)** and cDNA **(B)**. The colors indicate the different pH, while the shapes indicate to each phase the sample belongs.

As the applied decreases of the HRT occurred simultaneously with increases of the average loading rate, the effects of each of these individual parameters on the microbial community could not be distinguished. The CAP plots (Figure 5) for both DNA and cDNA showed, however, that sample separation, and thus microbial community structure, was dependent on pH and HRT/loading rate, and that the four operational phases identified were grouped separately.

## Discussion

To our knowledge, this is the first report of pilot-scale AD as a technology to treat real dairy processing wastewaters, *in situ*, at ambient temperatures. Good COD removals were obtained during most of the trial period; on average, soluble COD removals were above 80% during all phases, while total COD removal efficiency ranged from 50 to 70%. These results are comparable with those reported from laboratory-scale studies of low temperature AD to treat dairy wastewaters at laboratory-scale, indicating a successful scale up of the process. For example, an expanded granular sludge bed-anaerobic filter (EGSB-AF) treating synthetic skimmed dairy wastewater, operated at 10°C, presented removals of 74 to 90% (Bialek et al., 2013). Another study using two EGSB bioreactors operated at 15°C and treating real dairy wastewaters showed removals of 54 to 92% (Gunnigle et al., 2015a). The same reactors were initially operated at 37°C and exhibited comparable removals (88–96%). This indicates that the psychrophilic and mesophilic treatments of dairy wastewaters can have similar efficiencies for COD removals at low-medium organic loading rates.

The efficiency of the reactor performance, evidenced by the good COD removals reported, and a high methane content in the biogas, was underpinned by a stable core archaeal community. In particular, there was a high relative abundance of active methanogenic species of *Methanothrix*, which represented up to 50% of the active microbial community and up to 25.5% of the total community. The presence in high abundance of *Methanothrix* species in low-temperature AD systems was observed at laboratory-scale in several studies (Enright et al., 2009; Siggins et al., 2011a,b; Bandara et al., 2012; Gunnigle et al., 2015a,b; Keating et al., 2018). Furthermore, real-time PCR results of an archaeal populations revealed that *Methanosaeataceae* was the dominant methanogen in the bioreactor treating dilute dairy wastewater and its numbers remained stable during the complete trial (Bialek et al., 2013), along with high numbers of *Methanobacteriales* and *Methanomicrobiales*. Members of these orders were also found in the community in the pilot-scale reactor, but in low relative abundances. *Methanothrix* species are described to play an important role in the formation and maintenance of a strong granular sludge (MacLeod et al., 1990; McHugh et al., 2005) and it is believed to be dominant at low acetate concentrations (De Vrieze et al., 2012), but it was also recently reported to be able to become dominant at high acetate concentrations (Chen and He, 2015; Chen et al., 2017). *Methanobacteriaceae* species members could be found in total community, but their presence in the activity community was very low (~1%). These combined findings led us to believe that aceticlastic methanogenesis was the main active pathway for methane formation in in the pilot-scale reactor.

While DNA-based relative abundances indicates which microorganisms are present, relative abundances based on cDNA are a more accurate indicator of which populations are active at a given time point. In general, we observed a good agreement between both sets of data. The results obtained provided insights on the evolution and dynamics of the microbial community over time, and as result of reactor perturbations. For example, *Carnobacteriaceae* (mainly the genus *Trichoccocus*) family members were abundant in the beginning, both in total and active community, but their abundance decreased from ~13% (cDNA level) at day 0 to <1% at day 47. On the other hand, members of the family *Desulfobulbaceae* presented relative abundances of ~23% at day 0 in the active communit, but represented only 3% of the total community. Furthermore, their presence at cDNA level decreased to <2% after day 96. These results might indicate that the members of those families played important roles in the seed sludge, or source reactor, and for that, were abundant in the active community. However, these roles were less relevant or less well-suited to growth under the conditions prevailing in the pilot reactor, leading to their disappearance, both in the total and active communities. It was also observed that *Pseudomonas* species were not abundant at day 0, but their abundance in the active community increased during the start-up (phase I), remaining stable in the total community. While they were almost undetectable during phase II, they emerged again after the pH shock at day 221 (up to 20% in the total community and up to 54% in the active community), and decreased in relative abundances, both total and active, again when the reactor performance stabilized. This could indicate that they might have a competitive advantage when perturbations are induced to the reactor. *Pseudomonas* species have been identified as key players in AD (Shah et al., 2014) and it is possible that their versatility provided the necessary functional redundancy that allowed the reactor to stabilize after each perturbation. Members of *Rhodocyclaceae* family, mainly from the genera *Thauera* and *Azoarcos*, emerged during phase III. This family comprise mostly aerobic or denitrifying aquatic bacteria with versatile metabolisms (Wongwilaiwalin et al., 2010). They are also known to use acetate under anaerobic conditions (Wongwilaiwalin et al., 2010), which could lead to competition between them and the aceticlastic methanogens.

The most abundant members in the cDNA- and DNA-based bacterial community profile were unclassified members of Bacteroidetes' class vadinHA17, until end of phase II. On the other hand, during phases III and IV, other members of the Bacteroidetes phylum emerged in both communities, Bacteroidales ML635J-40, which increased from <2% until day 221 to ~10% at day 252, in both active and total communities, and remaining stable after that. This group was identified as being responsible for the hydrolysis of algae during anaerobic digestion at high pH (pH 10; Nolla-Ardèvol et al., 2015). Moreover, this group was identified as one of the more abundant inside submarine ikaite columns, a permanently cold (<6°C) and alkaline (pH >10) environment (Glaring et al., 2015). Those results seem to indicate an adaption of this group to alkaline environments and may explain why they emerged following the pH shocks in our reactor. Bacteroidetes are commonly found in the microbial communities of anaerobic digesters (Werner et al., 2011; Shah et al., 2014; Guo et al., 2015; Sun et al., 2015), including low-temperature AD digesters (McKeown et al., 2009; Abram et al., 2011; Bialek et al., 2012, 2013), and low-temperature AD digeste treating dairy wastewater (Bialek et al., 2011, 2014; Keating et al., 2018), which indicates their crucial role in anaerobic treatment. Their presence in abundance indicates a high hydrolytic activity in the system (Shah et al., 2014). Hydrolysis is a crucial step in AD systems and is often reported as the limiting step and the cause of poor reactor performances especially at lower temperatures, therefore a high abundance in the system is core to the efficiency of the process/system (Ma et al., 2013; Bialek et al., 2014; Azman et al., 2015). Their presence in high abundance in our system can be linked with the good reactor performance observed. Our results also showed a stable presence of active members of the *Synergistaceae* family. This family belongs to phylum Synergistetes, which was also observed in other reactors treating dairy wastewater (Gunnigle et al., 2015a; Keating et al., 2018; Callejas et al., 2019) and it is known to degrade peptides, proteins and amino acids. On the other hand, contrary to other studies of room/low temperature reactors treating dairy wastewater (Bialek et al., 2011, 2014; Keating et al., 2018; Callejas et al., 2019), our results showed very little active presence of Firmicutes. Nevertheless, Gunnigle et al. (2015a) reported a decreased in Firmicutes associated with low temperature. Interestingly, Callejas et al. (2019) observed an increase in Firmicutes from 29 to 79% after a pH increase in a full-scale UASB treating dairy wastewater.

On the other hand, the phylum Proteobacteria, commonly found in dairy-treating reactors (Bialek et al., 2011, 2014; Gunnigle et al., 2015a; Keating et al., 2018; Callejas et al., 2019), aside from *Pseudomonas*, represented 8 to 16% of the active community, although no family was highly abundant. These values are much lower than the 62% relative abundance of this phylum observed by Gunnigle et al. (2015a), but more similar to the 27% observed by Callejas et al. (2019). Overall, our results and the literature indicate that a high relative abundance of methanogens, specially *Methanotrix* species, and bacterial members of Bacteroidetes, *Synergistaceae* and Proteobacteria are the core players in active communities of AD-digesters treating dairy wastewater at low temperature. However, the relative abundances of the bacterial members is variable, most likely due to the differences in processes and products that can be found in this type of industry.

One of the known challenges inherent to the treatment of dairy wastewater is the presence of FOG. In this trial the influent FOG concentration to the pilot-reactor varied during the trial (21 to 244 mg/L). Although FOG are reported to benefit biogas production, they are also reported to cause operational challenges related to inhibition, substrate and product transport limitations, sludge floating, foaming and clogging biogas collection systems (Long et al., 2012). The recommended concentration of FOG for the optimal performance of the reactor was reported as being c. 100 mg/L (Passeggi et al., 2012); this value was exceeded for short periods of time during this trial, but did not result in any obvious effect on the performance or microbial community of the reactor. Furthermore, no clogging issues arose, and the presence of fat was not observed on the sludge granules.

The capacity to operate at low HRT is advantageous because it allows the reduction of reactor volumes, which in turn reduces the capital investment cost. In our trial, the HRT was reduced from 36 to 6 h in several steps over the trial. At the same time, an increase in the average loading rate was applied, and coincided with increased influent COD concentrations during the seasonal processing cycle. None of the changes HRT or loading rate, had a negative impact on the reactor performance in terms of either COD removal or biogas methane content. On the other hand, these changes could be correlated with changes in the microbial community. No other measured parameter could be tied to these community changes. This is the first major long-term study that describes such a clear correlation and suggests a role the HRT and loading rate in selecting the microbial population in granular sludge reactors. In the past, the effect of both parameters on the microbial community was studied separately, but even in this case, the literature is scarce. For example, it was shown that HRT had a role in selecting the microbial population and had an impact on the reactor performance of a UASB reactor treating synthetic wastewater with trichloroethylene (Zhang et al., 2015). The authors observed that the relative abundance of the different phyla, especially for the dominant phyla, changed with the different HRTs tested. The impact of a HRT change from 8 to 4 h was analyzed in an anaerobic moving bed membrane bioreactor fed with synthetic domestic wastewater (Win et al., 2016). In this case, both the microbial community and biogas production were affected by the variation in HRT, but not the COD removal efficiency. When HRT was reset to 8 h, the reactor performance was able to recover. The effect of increasing loading rate was analyzed in a UASB reactor treating diluted pharmaceutical fermentation wastewater by Chen et al. (2014), who reported a shift in the microbial community where Firmicutes, Bacteroidetes, Thermoplasmata and Methanobacteria became the dominant phyla at high organic loading rate (OLR).

pH is known to be a key parameter influencing microbial community composition and function (Liu et al., 2002; Zhang et al., 2016a,b). During this study, the pH was controlled to 7.5, but due to operational issues, occasional perturbations occurred. Both reactor performance and the microbial community structure were immediately impacted by these pH changes. Anaerobic reactions are highly pH dependent and the optimal pH for methane production should range between 6.5 and 7.5 (de Mes et al., 2003). However, a stable performance, with concomitant biogas production, might be achieved over a wider pH range (6.0–8.0). At pH values below 6.0 and above 8.3, inhibition of methanogens can occur (de Lemos Chernicharo, 2007). In our pilot reactor system, a decrease in COD removal efficiency and a shift in the microbial community was observed every time the pH increased above 8.5. Nevertheless, a decrease in the relative abundances of Archaea, and a consequent decrease in methane content in the gas, was only observed when the pH remained above this value for 4 days (days 246–251). The influence of pH on a microbial community was also observed in a staged anaerobic digestion system treating food waste, where it was one of the parameters responsible differences in the bacterial community (Gaby et al., 2017). Furthermore, a decrease of 95% of average specific methane yield and a corresponding decrease in the abundances of *Methanosarcina* and *Methanothrix* was observed at pH 8.5 in a two-phase anaerobic co-digestion of pig manure with maize Straw (Zhang et al., 2016b). Also, in other environments, such as soil, pH was reported to be one of the main factors responsible for shaping the microbial community (Bartram et al., 2014; Wu et al., 2017). Despite the microbial community changes, the pilot reactor performance was always able to recover, with efficient wastewater treatment performance (high methane content, good COD removal). In similar fashion, a full-scale UASB treating dairy wastewater suffered from a pH increase to 9.0 for 2 days (Callejas et al., 2019). The authors observed an effect on reactor performance, as well as a decrease in relative abundance for most phyla, but, also in this case, the reactor and community were able to recover from the pH imbalance.

It is known that, in response to a disturbance, the microbial community can either maintain the composition (resistance), temporarily change the composition, but returning to the initial one (resilience) or shift to a new composition able to perform identical processes (functional redundancy) (Allison and Martiny, 2008; Shade et al., 2012). In our system, each change in the HRT/loading rate or pH shock led the community to change to a different composition, but the performance of the system remained stable, which means that the main microbial functions were unaffected. These results point to functional redundancy in the sludge community, such as the switch between members of the Bacteroidetes family (unclassified vandinHa17 by *Rhodocyclaceae*) or the increase in abundance of *Pseudomonas* species when there were major perturbations in the reactor. Furthermore, it also points to some resistance since the main active core remained stable for most perturbations. A similar result was observed for the microbial communities of AD digesters treating molasses wastewater and disturbed with high salinity (De Vrieze et al., 2017). Such results pinpoint the importance of the microbiology for the success of the AD. Functional redundancy, resilience, and resistance in anaerobic sludge are fundamental for having a robust and versatile system, able to keep high performance standards even when facing wastewater variability and perturbations.

## Conclusions

The results obtained from this *in situ* pilot-scale trial represent a successful scale-up of ambient temperature AD as a technology with the ability to sustainably treat dairy-processing wastewaters at high-rate, resulting in high COD removal and high-quality biogas production. We have demonstrated the impact which operational parameters, such as pH and HRT/loading rate, have on system performance and/or microbial community composition. Notably, despite alterations to its composition, the microbial community was able to recover and perform up to a similar standard as before perturbations, thereby exhibiting clear hallmarks of functional redundancy, but also resistance by the main active archaeal core, who remained stable for most of the trial.

## Data Availability Statement

The raw data supporting the conclusions of this article will be made available by the authors, without undue reservation, to any qualified researcher.

## Author Contributions

LP, JC-A, and VO'F designed the experiment. LP and JC-A performed the experiments, analyzed the data, and wrote the manuscript. JR-G did the Bioinformatics and statistical analysis. JE-P helped with the reactor's maintenance and with the analytical methods. DH helped the reactor's performance analysis and commented on the manuscript. TM, MM, PW, and VO'F critically revised the manuscript. All authors read and approved the final manuscript.

### Conflict of Interest

DH and MM were employed by the company NVP Energy Ltd. The remaining authors declare that the research was conducted in the absence of any commercial or financial relationships that could be construed as a potential conflict of interest.

## Supplementary Material

Figure S1 Schematic representation of the pilot reactor.

Figure S2 Pilot reactor on site.

Figure S3 Temperature variation inside the reactor during the trial.

Figure S4 Heat-map of relative abundances for the relevant taxanomic groups.

Table S1 OTU count for DNA and cDNA.

Table S2 Primer coverage for Archaea and Bacteria.

## References

[B1] Abram F. Enright A. M. O'Reilly J. Botting C. H. Collins G. O'Flaherty V. (2011). A metaproteomic approach gives functional insights into anaerobic digestion. J. Appl. Microbiol. 110, 1550–1560. 10.1111/j.1365-2672.2011.05011.x 21447011

[B2] Akila G. Chandra T. S. (2007). Performance of an UASB reactor treating synthetic wastewater at low-temperature using cold-adapted seed slurry. Proc. Biochem. 42, 466–471. 10.1016/j.procbio.2006.09.010

[B3] Allison S. D. Martiny J. B. H. (2008). Resistance, resilience, and redundancy in microbial communities. Proc. Natl. Acad. Sci. U.S.A. 105(Suppl. 1):11512. 10.1073/pnas.0801925105 18695234 PMC2556421

[B4] APHA (2005). Standard Methods for the Examination of Water and Wastewater. American Public Health Association.

[B5] Azman S. Khadem A. F. van Lier J. B. Zeeman G. Plugge C. M. (2015). Presence and role of anaerobic hydrolytic microbes in conversion of lignocellulosic biomass for biogas production. Crit. Rev. Environ. Sci. Technol. 45, 2523–2564. 10.1080/10643389.2015.1053727

[B6] Bandara W. M. K. R. T. W. Kindaichi T. Satoh H. Sasakawa M. Nakahara Y. Takahashi M.. (2012). Anaerobic treatment of municipal wastewater at ambient temperature: analysis of archaeal community structure and recovery of dissolved methane. Water Res. 46, 5756–5764. 10.1016/j.watres.2012.07.061 22921025

[B7] Bartram A. K. Jiang X. Lynch M. D. J. Masella A. P. Nicol G. W. Dushoff J.. (2014). Exploring links between pH and bacterial community composition in soils from the Craibstone Experimental Farm. FEMS Microbiol. Ecol. 87, 403–415. 10.1111/1574-6941.12231 24117982

[B8] Bialek K. Cysneiros D. O'Flaherty V. (2013). Low-temperature (10°C) anaerobic digestion of dilute dairy wastewater in an EGSB bioreactor: microbial community structure, population dynamics, and kinetics of methanogenic populations. Archaea 2013:10. 10.1155/2013/346171 24089597 PMC3780618

[B9] Bialek K. Cysneiros D. O'Flaherty V. (2014). Hydrolysis, acidification and methanogenesis during low-temperature anaerobic digestion of dilute dairy wastewater in an inverted fluidised bioreactor. Appl. Microbiol. Biotechnol. 98, 8737–8750. 10.1007/s00253-014-5864-7 24946864

[B10] Bialek K. Kim J. Lee C. Collins G. Mahony T. O'Flaherty V. (2011). Quantitative and qualitative analyses of methanogenic community development in high-rate anaerobic bioreactors. Water Res. 45, 1298–1308. 10.1016/j.watres.2010.10.010 21093011

[B11] Bialek K. Kumar A. Mahony T. Lens P. N. L. O' Flaherty V. (2012). Microbial community structure and dynamics in anaerobic fluidized-bed and granular sludge-bed reactors: influence of operational temperature and reactor configuration. Microb. Biotechnol. 5, 738–752. 10.1111/j.1751-7915.2012.00364.x 22967313 PMC3815895

[B12] Callejas C. Fernández A. Passeggi M. Wenzel J. Bovio P. Borzacconi L.. (2019). Microbiota adaptation after an alkaline pH perturbation in a full-scale UASB anaerobic reactor treating dairy wastewater. Bioproc. Biosyst. Eng. 42, 2035–2046. 10.1007/s00449-019-02198-3 31506821

[B13] Caporaso J. G. Lauber C. L. Walters W. A. Berg-Lyons D. Huntley J. Fierer N.. (2012). Ultra-high-throughput microbial community analysis on the Illumina HiSeq and MiSeq platforms. ISME J. 6, 1621–1624. 10.1038/ismej.2012.8 22402401 PMC3400413

[B14] Chen S. Cheng H. Liu J. Hazen T. C. Huang V. He Q. (2017). Unexpected competitiveness of Methanosaeta populations at elevated acetate concentrations in methanogenic treatment of animal wastewater. Appl. Microbiol. Biotechnol. 101, 1729–1738. 10.1007/s00253-016-7967-9 27858134

[B15] Chen S. He Q. (2015). Persistence of Methanosaeta populations in anaerobic digestion during process instability. J. Indust. Microbiol. Biotechnol. 42, 1129–1137. 10.1007/s10295-015-1632-7 25956380

[B16] Chen Z. Wang Y. Li K. Zhou H. (2014). Effects of increasing organic loading rate on performance and microbial community shift of an up-flow anaerobic sludge blanket reactor treating diluted pharmaceutical wastewater. J. Biosci. Bioeng. 118, 284–288. 10.1016/j.jbiosc.2014.02.027 24725962

[B17] de Lemos Chernicharo C. A. (2007). Anaerobic Reactors. London: IWA Publishing.

[B18] de Mes T. Z. D. Stams A. J. M. Reith J. H. Zeeman G. (2003). Methane production by anaerobic digestion of wastewater and solid wastes, in Bio-Methane and Bio-Hydrogen: Status and Perspectives of Biological Methane and Hydrogen Production, eds Reith J. H. Wijffels R. H. Barten H. (The Haggue: Dutch Biological Hydrogen Foundation), 58–101.

[B19] De Vrieze J. Christiaens M. E. R. Walraedt D. Devooght A. Ijaz U. Z. Boon N. (2017). Microbial community redundancy in anaerobic digestion drives process recovery after salinity exposure. Water Res. 111, 109–117. 10.1016/j.watres.2016.12.042 28063283

[B20] De Vrieze J. Hennebel T. Boon N. Verstraete W. (2012). Methanosarcina: the rediscovered methanogen for heavy duty biomethanation. Bioresour. Technol. 112, 1–9. 10.1016/j.biortech.2012.02.079 22418081

[B21] Demirel B. Yenigun O. Onay T. T. (2005). Anaerobic treatment of dairy wastewaters: a review. Proc. Biochem. 40, 2583–2595. 10.1016/j.procbio.2004.12.015

[B22] Elmitwalli T. A. Oahn K. L. T. Zeeman G. Lettinga G. (2002). Treatment of domestic sewage in a two-step anaerobic filter/anaerobic hybrid system at low temperature. Water Res. 36, 2225–2232. 10.1016/S0043-1354(01)00438-9 12108715

[B23] Elmitwalli T. A. Zandvoort M. H. Zeeman G. Bruning H. Lettinga G. (1999). Low temperature treatment of domestic sewage in upflow anaerobic sludge blanket and anaerobic hybrid reactors. Water Sci. Technol. 39, 177–185. 10.2166/wst.1999.0237

[B24] Enright A.-M. McGrath V. Gill D. Collins G. O'Flaherty V. (2009). Effect of seed sludge and operation conditions on performance and archaeal community structure of low-temperature anaerobic solvent-degrading bioreactors. Syst. Appl. Microbiol. 32, 65–79. 10.1016/j.syapm.2008.10.003 19108975

[B25] Gaby J. C. Zamanzadeh M. Horn S. J. (2017). The effect of temperature and retention time on methane production and microbial community composition in staged anaerobic digesters fed with food waste. Biotechnol. Biofuels 10:302. 10.1186/s13068-017-0989-4 29255485 PMC5729454

[B26] Gil-Pulido B. Tarpey E. Almeida E. L. Finnegan W. Zhan X. Dobson A. D. W.. (2018). Evaluation of dairy processing wastewater biotreatment in an IASBR system: aeration rate impacts on performance and microbial ecology. Biotechnol. Rep. 19:e00263. 10.1016/j.btre.2018.e00263 29992097 PMC6036646

[B27] Glaring M. A. Vester J. K. Lylloff J. E. Al-Soud W. A. Sorensen S. J. Stougaard P. (2015). Microbial diversity in a permanently cold and alkaline environment in Greenland. PLoS ONE 10:e0124863. 10.1371/journal.pone.0124863 25915866 PMC4411134

[B28] Griffiths R. I. Whiteley A. S. O'Donnell A. G. Bailey M. J. (2000). Rapid method for coextraction of DNA and RNA from natural environments for analysis of ribosomal DNA- and rRNA-based microbial community composition. Appl. Environ. Microbiol. 66, 5488–5491. 10.1128/AEM.66.12.5488-5491.2000 11097934 PMC92488

[B29] Gunnigle E. Nielsen J. L. Fuszard M. Botting C. H. Sheahan J. O'Flaherty V.. (2015a). Functional responses and adaptation of mesophilic microbial communities to psychrophilic anaerobic digestion. FEMS Microbiol. Ecol. 91:fiv132. 10.1093/femsec/fiv132 26507125

[B30] Gunnigle E. Siggins A. Botting C. H. Fuszard M. O'Flaherty V. Abram F. (2015b). Low-temperature anaerobic digestion is associated with differential methanogenic protein expression. FEMS Microbiol. Lett. 362:fnv059. 10.1093/femsle/fnv059 25862577

[B31] Guo J. Peng Y. Ni B.-J. Han X. Fan L. Yuan Z. (2015). Dissecting microbial community structure and methane-producing pathways of a full-scale anaerobic reactor digesting activated sludge from wastewater treatment by metagenomic sequencing. Microb. Cell Fact. 14:33. 10.1186/s12934-015-0218-4 25880314 PMC4381419

[B32] Hughes D. Enright A. M. Mahony T. O' Flaherty V. (2011). Novel Anaerobic Sewage Treatment and Bioenergy Production: High-Rate Anaerobic Digestion as a Core Technology for Sustainable Treatment of Municipal and Low-Strength Industrial Wastewaters. Environmental Protection Agency.

[B33] Keating C. Hughes D. Mahony T. Cysneiros D. Ijaz U. Z. Smith C. J.. (2018). Cold adaptation and replicable microbial community development during long-term low-temperature anaerobic digestion treatment of synthetic sewage. FEMS Microbiol. Ecol. 94:fiy095. 10.1093/femsec/fiy095 29846574 PMC5995215

[B34] Kembel S. W. Cowan P. D. Helmus M. R. Cornwell W. K. Morlon H. Ackerly D. D.. (2010). Picante: R tools for integrating phylogenies and ecology. Bioinformatics 26, 1463–1464. 10.1093/bioinformatics/btq166 20395285

[B35] Lateef A. Chaudhary M. N. Ilyas S. (2013). Biological treatment of dairy wastewater using actiavted sludge. Science Asia 39, 179–185. 10.2306/scienceasia1513-1874.2013.39.179

[B36] Liu W.-T. Chan O.-C. Fang H. H. P. (2002). Microbial community dynamics during start-up of acidogenic anaerobic reactors. Water Res. 36, 3203–3210. 10.1016/S0043-1354(02)00022-2 12188116

[B37] Long J. H. Aziz T. N. de los Reyes F. L. Ducoste J. J. (2012). Anaerobic co-digestion of fat, oil, and grease (FOG): a review of gas production and process limitations. Proc. Safety Environ. Protect. 90, 231–245. 10.1016/j.psep.2011.10.001

[B38] Ma J. Frear C. Wang Z.-W. Yu L. Zhao Q. Li X.. (2013). A simple methodology for rate-limiting step determination for anaerobic digestion of complex substrates and effect of microbial community ratio. Bioresour. Technol. 134, 391–395. 10.1016/j.biortech.2013.02.014 23489573

[B39] MacLeod F. A. Guiot S. R. Costerton J. W. (1990). Layered structure of bacterial aggregates produced in an upflow anaerobic sludge bed and filter reactor. Appl. Environ. Microbiol. 56, 1598–1607. 10.1128/AEM.56.6.1598-1607.1990 2383005 PMC184478

[B40] McHugh S. Collins G. Mahony T. Flaherty V. (2005). Biofilm reactor technology for low temperature anaerobic waste treatment: microbiology and process characteristics. Water Sci. Technol. 52:107 10.2166/wst.2005.0188

[B41] McHugh S. Collins G. O'Flaherty V. (2006). Long-term, high-rate anaerobic biological treatment of whey wastewaters at psychrophilic temperatures. Bioresour. Technol. 97, 1669–1678. 10.1016/j.biortech.2005.07.020 16168638

[B42] McIlroy S. J. Kirkegaard R. H. McIlroy B. Nierychlo M. Kristensen J. M. Karst S. M.. (2017). MiDAS 2.0: an ecosystem-specific taxonomy and online database for the organisms of wastewater treatment systems expanded for anaerobic digester groups. Database. 10.1093/database/bax016 28365734 PMC5467571

[B43] McKeown R. M. Hughes D. Collins G. Mahony T. O'Flaherty V. (2012). Low-temperature anaerobic digestion for wastewater treatment. Curr. Opin. Biotechnol. 23, 444–451. 10.1016/j.copbio.2011.11.025 22176749

[B44] McKeown R. M. Scully C. Mahony T. Collins G. O'Flaherty V. (2009). Long-term (1243 days), low-temperature (4–15°C), anaerobic biotreatment of acidified wastewaters: bioprocess performance and physiological characteristics. Water Res. 43, 1611–1620. 10.1016/j.watres.2009.01.015 19217137

[B45] McMurdie P. J. Holmes S. (2013). phyloseq: an R package for reproducible interactive analysis and graphics of microbiome census data. PLoS ONE 8:e61217. 10.1371/journal.pone.0061217 23630581 PMC3632530

[B46] Nolla-Ardèvol V. Strous M. Tegetmeyer H. E. (2015). Anaerobic digestion of the microalga Spirulina at extreme alkaline conditions: biogas production, metagenome, and metatranscriptome. Front. Microbiol. 6:597. 10.3389/fmicb.2015.00597 26157422 PMC4475827

[B47] Oksanen J. (2012). Constrained Ordination: Tutorial With R and Vegan. Available online at: https://www.mooreecology.com/uploads/2/4/2/1/24213970/constrained_ordination.pdf (accessed March, 2020)

[B48] O'Reilly J. Chinalia F. A. Mahony T. Collins G. Wu J. O'Flaherty V. (2009). Cultivation of low-temperature (15°C), anaerobic, wastewater treatment granules. Lett. Appl. Microbiol. 49, 421–426. 10.1111/j.1472-765X.2009.02682.x 19674296

[B49] Passeggi M. López I. Borzacconi L. (2012). Modified UASB reactor for dairy industry wastewater: performance indicators and comparison with the traditional approach. J. Clean. Prod. 26, 90–94. 10.1016/j.jclepro.2011.12.022

[B50] Quaiser J. Bitter E. (2016). Wastewater Treatment in the Dairy Processing Industry - Recovering Energy Using Anaerobic Technology. 10.13140/RG.2.1.3875.4965

[B51] Ramiro-Garcia J. Hermes G. D. A. Giatsis C. Sipkema D. Zoetendal E. Schaap P. (2018). NG-Tax, a highly accurate and validated pipeline for analysis of 16S rRNA amplicons from complex biomes [version 2; peer review: 2 approved, 1 approved with reservations, 1 not approved]. F1000Research 5:1791 10.12688/f1000research.9227.2 PMC6419982 30918626

[B52] Shade A. Peter H. Allison S. D. Baho D. L. Berga M. Bürgmann H.. (2012). Fundamentals of microbial community resistance and resilience. Front. Microbiol. 3:417. 10.3389/fmicb.2012.00417 23267351 PMC3525951

[B53] Shah A. F. Mahmood Q. Shah M. M. Pervez A. Asad S. A. (2014). Microbial ecology of anaerobic digesters: the key players of anaerobiosis. Sci. World J. 2014:21 10.1155/2014/183752 PMC3950365 24701142

[B54] Shah Y. (2014). Water for Energy and Fuel Production. Boca Raton, FL: CRC Press 10.1201/b16904

[B55] Siggins A. Enright A.-M. O'Flaherty V. (2011a). Low-temperature (7°C) anaerobic treatment of a trichloroethylene-contaminated wastewater: Microbial community development. Water Res. 45, 4035–4046. 10.1016/j.watres.2011.05.013 21664638

[B56] Siggins A. Enright A.-M. O'Flaherty V. (2011b). Temperature dependent (37–15°C) anaerobic digestion of a trichloroethylene-contaminated wastewater. Bioresour. Technol. 102, 7645–7656. 10.1016/j.biortech.2011.05.055 21715158

[B57] Slavov A. K. (2017). General characteristics and treatment possibilities of dairy wastewater – a review. Food Technol. Biotechnol. 55, 14–28. 10.17113/ftb.55.01.17.4520 28559730 PMC5434364

[B58] Sun L. Pope P. B. Eijsink V. G. H. Schnürer A. (2015). Characterization of microbial community structure during continuous anaerobic digestion of straw and cow manure. Microb. Biotechnol. 8, 815–827. 10.1111/1751-7915.12298 26152665 PMC4554469

[B59] Thorn C. E. Bergesch C. Joyce A. Sambrano G. McDonnell K. Brennan F.. (2018). A robust, cost-effective method for DNA, RNA and protein co-extraction from soil, other complex microbiomes, and pure cultures. Mol. Ecol. Resour. 19,439–455. 10.1111/1755-0998.12979 30565880

[B60] USEPA (2010). Method 1664, Revision B: n-Hexane Extractable Material (HEM; Oil and Grease) and Silica Gel Treated n-Hexane Extractable Material (SGT-HEM; Non-polar Material) by Extraction and Gravimetry. Washington, DC: United States Environmental Protection Agency. EPA-821-R-10-001.

[B61] Venkiteshwaran K. Bocher B. Maki J. Zitomer D. (2015). Relating anaerobic digestion microbial community and process function. Microbiol. Insights 8(Suppl 2), 37–44. 10.4137/MBI.S33593 27127410 PMC4841157

[B62] Werner J. J. Knights D. Garcia M. L. Scalfone N. B. Smith S. Yarasheski K.. (2011). Bacterial community structures are unique and resilient in full-scale bioenergy systems. Proc. Natl. Acad. Sci. U.S.A. 108, 4158–4163. 10.1073/pnas.1015676108 21368115 PMC3053989

[B63] Wickham H. (2016). ggplot2: Elegant Graphics for Data Analysis. New York, NY: Springer-Verlag.

[B64] Win T. T. Kim H. Cho K. Song K. G. Park J. (2016). Monitoring the microbial community shift throughout the shock changes of hydraulic retention time in an anaerobic moving bed membrane bioreactor. Bioresour. Technol. 202, 125–132. 10.1016/j.biortech.2015.11.085 26706726

[B65] Wongwilaiwalin S. Rattanachomsri U. Laothanachareon T. Eurwilaichitr L. Igarashi Y. Champreda V. (2010). Analysis of a thermophilic lignocellulose degrading microbial consortium and multi-species lignocellulolytic enzyme system. Enzyme Microb. Technol. 47, 283–290. 10.1016/j.enzmictec.2010.07.013

[B66] Wu Y. Zeng J. Zhu Q. Zhang Z. Lin X. (2017). pH is the primary determinant of the bacterial community structure in agricultural soils impacted by polycyclic aromatic hydrocarbon pollution. Sci. Rep. 7:40093. 10.1038/srep40093 28051171 PMC5209717

[B67] Zhang D. Zhu W. Tang C. Suo Y. Gao L. Yuan X. (2012). Bioreactor performance and methanogenic population dynamics in a low-temperature (5–18°C) anaerobic fixed-bed reactor. *Bioresour*. Technol. 104(Suppl. C), 136–143. 10.1016/j.biortech.2011.10.086 22137750

[B68] Zhang L. Ban Q. Li J. Jha A. K. (2016a). Response of syntrophic propionate degradation to ph decrease and microbial community shifts in an UASB reactor. J. Microbiol. Biotechnol. 26, 1409–1419. 10.4014/jmb.1602.02015 27160579

[B69] Zhang L. Zhang K. Gao W. Zhai Z. Lian J. Du L. (2016b). Influence of temperature and ph on methanogenic digestion in two-phase anaerobic co-digestion of pig manure with maize straw. J. Residuals Sci. Technol. 13, S27–S32. 10.12783/issn.1544-8053/13/S1/5

[B70] Zhang Y. Wang X. Hu M. Li P. (2015). Effect of hydraulic retention time (HRT) on the biodegradation of trichloroethylene wastewater and anaerobic bacterial community in the UASB reactor. Appl. Microbiol. Biotechnol. 99, 1977–1987. 10.1007/s00253-014-6096-6 25277413

