# Supplementary figures and images for: Microbial Community Redundancy and Resilience Underpins High-Rate Anaerobic Treatment of Dairy-Processing Wastewater at Ambient Temperatures
Source: Front Bioeng Biotechnol. 2020 Mar 13;8:192. doi: 10.3389/fbioe.2020.00192 (PMC7082317; doi:10.3389/fbioe.2020.00192)

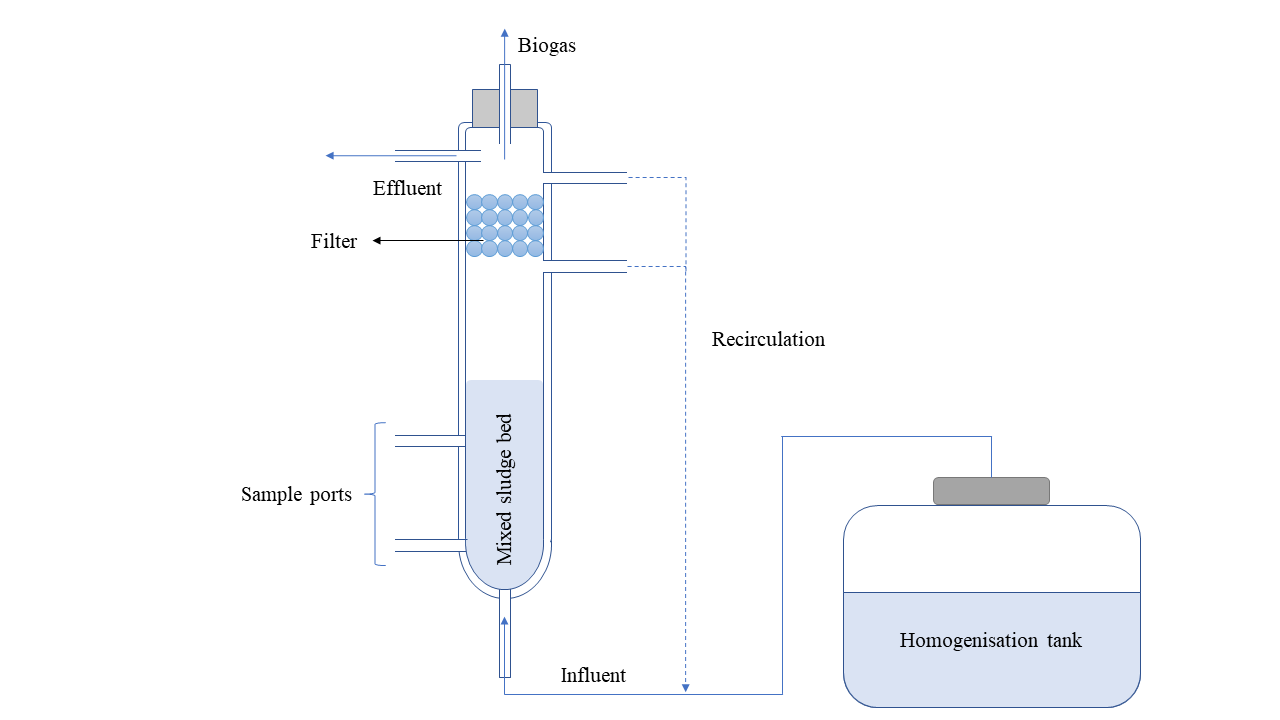

Supplement: Figure S1 — Schematic representation of the pilot reactor. [file Image_1.PNG]

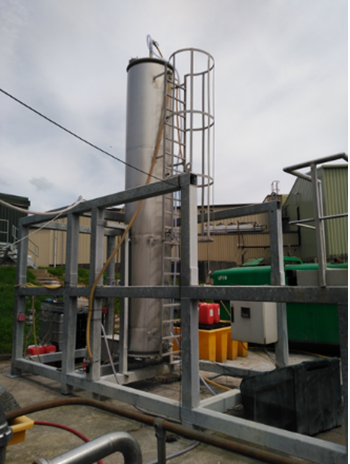

Supplement: Figure S2 — Pilot reactor on site. [file Image_2.TIF]

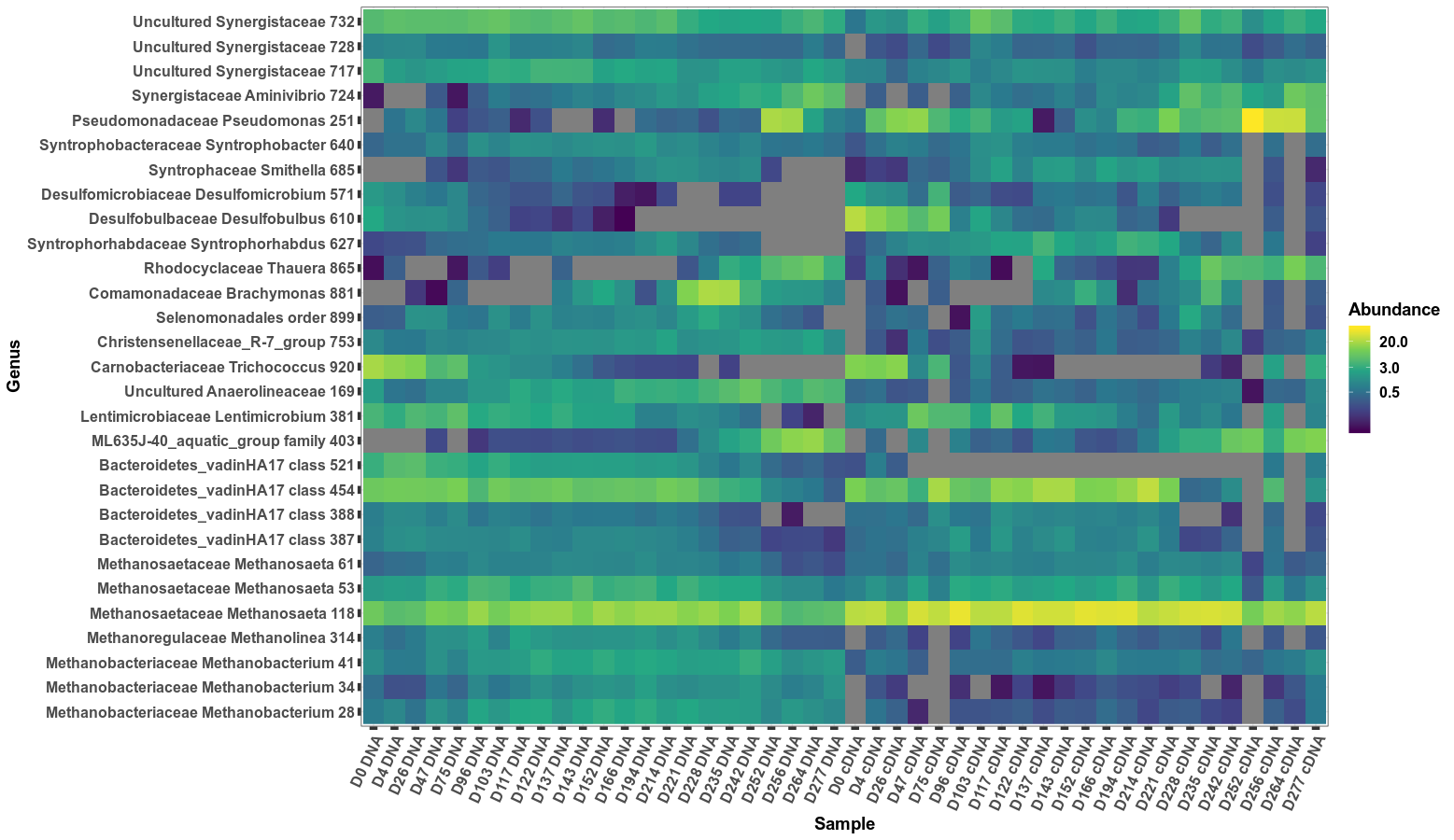

Supplement: Figure S4 — Heat-map of relative abundances for the relevant taxanomic groups. [file Image_4.TIFF]
